# Special Issue “New Drugs Regulating Cytoskeletons in Human Health and Diseases”

**DOI:** 10.3390/ijms27041911

**Published:** 2026-02-17

**Authors:** Gilles Breuzard, Maxime Robin, Hervé Kovacic

**Affiliations:** 1 Faculté des Sciences Médicales et Paramédicales, Institut de Neurophysiopathologie (INP), UMR 7051, CNRS, Aix Marseille Université, 13005 Marseille, France; herve.kovacic@univ-amu.fr; 2 Mediterranean Institute of Biodiversity and Marine and Continental Ecology, IRD, CNRS UMR7263, Aix-Marseille Université, 13013 Marseille, France; maxim.robin@univ-amu.fr

## 1. Introduction

Cytoskeletons are dynamic and multifunctional cellular frameworks composed of microtubules, actin filaments, and intermediate filaments. They orchestrate essential biological processes such as cell division and migration, intracellular transport, and signal transduction. Dysregulation of cytoskeletal architecture and signaling is implicated in a wide variety of human diseases, including cancer, neurodegenerative disorders, cardiovascular pathologies, and chemotherapy-induced neuropathies. Given that cytoskeletons are key components of eukaryotic cells, it is not surprising that they continue to represent a prime pharmacological target in human health and disease research.

This Special Issue of the *International Journal of Molecular Sciences*, “New Drugs Regulating Cytoskeletons in Human Health and Diseases”, brings together seven original and review articles that explore novel therapeutic strategies targeting cytoskeletal components. These contributions span diverse disease contexts—from glioblastoma and triple-negative breast cancer to heart failure and peripheral neuropathy—and highlight innovative small molecules, hybrid compounds, and mechanistic insights into cytoskeletal regulation.

## 2. Overview of Published Articles

Over the last five decades, Tau has been widely investigated in the context of adult neurodegenerative disorders, in which abnormal post-translational modifications, particularly hyperphosphorylation, disrupt its microtubule-stabilizing functions [1,2], ultimately leading to neuron toxicity. More recently, expanded research has revealed that aberrant Tau expression can also contribute to oncogenic processes, including those driving highly aggressive brain tumors such as glioblastoma (GBM) [3]. In contribution 1, Relave et al. investigate 2-aminothiazole–flavonoid hybrid derivatives [4] that bind Tau protein in glioblastoma (GBM) cells. Using 3D multicellular spheroids, they demonstrate that one compound significantly impairs proliferation and migration in Tau-expressing GBM cells. The results suggest that these hybrids may modulate Tau–microtubule interactions and influence PI3K/Akt and p53 signaling pathways. Notably, repeated treatment enhances compound bioavailability, supporting its potential as a therapeutic candidate for GBM. In conclusion, this compound deserves particular attention as a promising candidate for specifically targeting Tau-expressing cancers such as GBM.

Vékony et al. (contribution 2) explore the interaction between the neuropeptide pituitary adenylate cyclase-activating polypeptide (PACAP) and actin filaments using spectroscopic approaches. PACAP is a neuroprotective peptide that contributes to neuronal development, notably by affecting the actin cytoskeleton and neuron motility [5]. In this study, the authors explore the effect of PACAP on the modulation of actin organization, through its impact on filament assembly, and the molecular mechanisms involved. Based on their previously studied collection of PACAP-derived fragments [6,7], the authors identify PACAP38 and PACAP6-38 as the most effective peptides in promoting actin assembly. Although additional research will be required to clarify the physiological implications of these interactions, these observations reveal the potential therapeutic benefit of both peptides for neurodegenerative conditions or treatment following a neural injury.

The third article is from Mariotto et al. (contribution 3), who synthesize a new series of 2-aroyl benzofuran-based hydroxamic acids designed as dual tubulin–HDAC inhibitors. In recent years, efforts to improve anticancer efficacy have increasingly turned toward designing single molecules capable of acting on more than one cellular target. This strategy aims to bypass the shortcomings of conventional monotherapies and drug combinations by creating compounds that can simultaneously disrupt strong potential oncogenic multitargets. For example, researchers created a compound that could disturb both tubulin dynamics and histone deacetylase (HDAC) activity [8,9]. Here, they showed that many new synthesized methoxy-substituted 2-(3′,4′,5′-trimethoxybenzoyl)benzo[b]furan derivatives [10] exhibit potent antiproliferative activity against multiple cancer cell lines, outperforming combretastatin A-4 compounds 11a and 6g. These new compounds inhibit tubulin polymerization via colchicine-site binding, although HDAC6 inhibition remains limited. Notably, compound 6i emerges as a particularly strong candidate for subsequent structure-based optimization. Future design strategies should focus on refining the linker region to enhance its binding to HDAC active sites, while maintaining robust interaction with the colchicine-binding region of tubulin.

Li et al. (contribution 4) examine how the TRPV1 and TRPM8 ion channels contribute to the development of paclitaxel-induced peripheral neuropathic pain (PIPNP). Chemotherapy-induced peripheral neuropathy is a major dose-limiting complication of anticancer treatment and can arise during therapy and even persist long after it has ended [11,12]. Affected patients frequently experience sensory disturbances in a characteristic distal pattern, often described as a “glove-and-stocking” distribution, accompanied by burning sensations, tingling, numbness, or heightened pain sensitivity in the extremities. Here, the authors demonstrate that TRPV1 is upregulated in dorsal root ganglion (DRG) neurons during PIPNP, contributing to mechanical hyperalgesia. Conversely, TRPM8 expression and activity are reduced. These findings show that activation of TRPM8 via menthol or WS-12 attenuates pain and inhibits TRPV1 function, suggesting a novel analgesic mechanism based on TRP channel crosstalk.

The widespread occurrence of breast cancer and its propensity to develop drug resistance highlight the need for a comprehensive understanding of the molecular mechanisms involved [13]. In the fifth article, Dugina et al. (contribution 5) investigate the intricate pathways associated with secondary resistance to taxol in triple-negative breast cancer (TNBC) cells, with a particular focus on the changes observed in the cytoplasmic actin isoforms. Their previous findings show that β-actin depletion and a reciprocal γ-actin increase promoted chromosomal instability in the TNBC cell model [14]. Here, they report a shift toward γ-actin predominance, associated with increased motility, invasiveness, and epithelial-to-mesenchymal transition. These changes are accompanied by altered tubulin isotype expression and microtubule reorganization. Moreover, a transcriptomic approach reveals upregulation of genes involved in migration-, adhesion-, and actin-based processes, highlighting the role of actin isoform balance in drug resistance and metastasis. Prolonged treatment with taxol, accompanied by the development of resistance to the drug, appears to drive the TNBC cell line toward a more aggressive, metastasis-prone phenotype.

Diastolic heart failure, commonly referred to as heart failure with preserved ejection fraction (HFpEF), represents a multifaceted cardiac disorder that remains increasingly prevalent [15]. At the cellular level, disruptions in cytoskeletal organization combined with impaired mitochondrial function are commonly observed and contribute to the pathophysiology of impaired relaxation. Therefore, it is essential to understand the mechanisms beneath elevated diastolic filling pressures so that new therapeutic strategies can be developed. In their review (contribution 6), Er et al. delve into the role of cytoskeletal disorganization in HFpEF. They identify five therapeutic targets: extracellular matrix, costamere, ion channels, sarcomere, and mitochondria. The authors suggest that improving the predictive value of existing therapies will require more sophisticated preclinical models that more accurately mirror the multifaceted biology of HFpEF. They also emphasize that the therapeutic benefit could be increased by combining agents that act on several interconnected pathways, such as the extracellular matrix, cytoskeletal organization, and mitochondrial function [16], which could ultimately strengthen treatment responses and facilitate their successful translation into clinical practice.

The sixth text published in this Special Issue is from Ezzo and Etienne-Manneville, which presents a comprehensive review of microtubule-targeting agents (MTAs) with applications in brain pathologies such as gliomas and neurodegenerative disorders [17,18]. The authors outline the characterization of nine separate ligand-binding regions on tubulin, spanning well-established sites, such as those targeted by taxanes, vinca-alkaloids, and colchicine, and more recently identified pockets bound by agents (tumabulin and pironetin). By integrating recent progress in structural biology and medicinal chemistry, the authors demonstrate how these insights are driving the development of new MTAs with functions that extend beyond classical anti-mitotic activity. They also discuss how MTAs can be combined with other therapeutic modalities, such as radiotherapy, HDAC inhibition or antibody–drug conjugates, to achieve synergistic effects, particularly in GBM models. Altogether, the authors present MTAs as increasingly versatile molecules whose future optimization will aim to enhance therapeutic potency, minimize adverse effects, and counteract resistance mechanisms in diseases affecting the brain.

## 3. Conclusions

This Special Issue underscores the central role of the cytoskeleton in diverse pathological processes and highlights the therapeutic potential of targeting cytoskeletal components. The featured studies span a wide range of approaches, including small-molecule inhibitors, hybrid compounds, neuropeptides, and ion channel modulators. Multiple contributions emphasize the importance of advanced disease models—such as 3D spheroids and in vivo systems—for evaluating drug efficacy and mechanisms of action.

The translational relevance of cytoskeletal-targeting agents is particularly evident in complex diseases like GBM, TNBC, and HFpEF, where cytoskeletal dysfunction intersects with genetic, metabolic, and biomechanical factors. Future research should prioritize the development of compounds with improved selectivity, BBB permeability, and safety profiles. Moreover, the integration of omics technologies and personalized medicine approaches will be essential for identifying the patient subgroups that are most likely to benefit from cytoskeletal modulation.

This collection of papers serves as a platform for advancing cytoskeletal pharmacology and encourages further exploration into the therapeutic modulation of cellular architecture across diverse clinical contexts.

## Data Availability

Data are available upon request.
